# Environmental and Economic LCA Comparison of Flexural Strengthening Solutions for a Reinforced Concrete Beam

**DOI:** 10.3390/ma17235879

**Published:** 2024-11-30

**Authors:** Pedro Frazão Pedroso, João R. Correia, José D. Silvestre, João P. Firmo, Mário Garrido

**Affiliations:** Civil Engineering Research and Innovation for Sustainability (CERIS), Department of Civil Engineering, Architecture and Environment, Instituto Superior Técnico, University of Lisbon, 1049-001 Lisbon, Portugal; pedroefpedroso@tecnico.ulisboa.pt (P.F.P.); joao.ramoa.correia@tecnico.ulisboa.pt (J.R.C.); jfirmo@civil.ist.utl.pt (J.P.F.); mgarrido@civil.ist.utl.pt (M.G.)

**Keywords:** LCA, sustainability, strengthening, concrete, jacketing, CFRP strips, steel plate

## Abstract

The construction sector is one of the largest creators and distributors of wealth, contributing to economic growth worldwide. However, this economic growth comes together with very high environmental impacts. Thus, rehabilitation solutions that can adapt the current building stock to today’s structural requirements are needed, increasing structural safety, while avoiding the production of demolition waste and the extraction of virgin raw materials, hence lowering the construction sector’s environmental impacts. Such rehabilitation solutions need to be environmentally and economically sound so that stakeholders can make informed decisions based on their needs and priorities. This paper presents a case study of an existing reinforced concrete beam, whose flexural resistance is increased using four alternative strengthening solutions: concrete jacketing, without and with increasing the cross-section size, and plate bonding, using either carbon fibre-reinforced polymer (CFRP) strips or steel plates. These solutions are studied via an environmental and economic cradle-to-gate life cycle assessment (LCA), resulting in a comprehensive comparison of their environmental and economic impacts, followed by a multicriteria and sensitivity analysis and eco-cost approach to determine the optimal solution. According to the criteria considered in the study, when environmental impacts are more valued, the concrete jacketing solution presents the best results and, when cost is dominant in the decision, the bonding of CFRP strips becomes the optimal solution.

## 1. Introduction

The construction sector is facing several challenges that are becoming central to public policy, namely the concern with the depletion of natural resources or waste generation [1,2,3]. In this respect, this sector is responsible for up to 36% of the waste generated worldwide [1], this figure being 25% to 30% in the European Union (EU) [3]. About 50% of the global steel production is used for construction, and cement production contributes to 7% of the total greenhouse gas (GHG) emissions [4].

Considering that 85% of all buildings in the EU were built before 2001 and between 85% and 95% of the buildings in use today will still be in service by 2050 [5], the idea of renovating buildings, especially to comply with new structural standards and eventual changes in use of buildings, is of paramount importance.

Older buildings may not present the structural performance required today, as the evolution of materials and methods leads to updates of building codes [5]. This means that the safety of older buildings needs to be increased to meet current applicable regulations; this is the case of the seismic resistance in many countries, namely in Portugal, in which even reinforced concrete (RC) buildings built before 1983 generally present insufficient structural performance [6]. Another frequent situation that leads to insufficient performance is changes of use in buildings, such as those involving additional dead loads or live loads. Whatever the case, structural strengthening of structural members is often necessary.

Several strengthening solutions can be used to improve the resistance of structural members in buildings. In the case of RC members, these strengthening solutions include: (i) more intrusive techniques that require the partial demolition of the member to allow the introduction of additional reinforcement and/or, if necessary, increasing the area of the cross-section (jacketing), using either conventional concrete [7] or (ultra-)high-performance concrete (UHPC) [8,9]; and (ii) non-destructive techniques, in which new reinforcement, such as fibre-reinforced polymer (FRP) composite strips or sheets [10,11] or steel plates [12], is bonded to the surface(s) of the RC members. It is important to study these structural strengthening solutions from a life cycle assessment (LCA) perspective, not only to minimize the need to demolish old buildings and replace them with new construction but also to provide stakeholders with information about the environmental and economic impacts of the available options.

This paper presents the case study of an RC beam as a reference to assess the environmental and economic performance of four different flexural strengthening solutions commonly used in civil engineering practice: (i) jacketing by adding steel rebars keeping the concrete cross-section unchanged; (ii) jacketing by adding steel rebars and increasing the concrete cross-section; (iii) bonding a steel plate, and (iv) bonding carbon-FRP (CFRP) strips. These solutions are studied both from economic and environmental standpoints, namely regarding their environmentally harmful emissions.

## 2. Literature Review

The strengthening solutions mentioned above, although well known, lack research from an environmental and economic standpoint. Previous studies on strengthening solutions used in civil engineering were found to have addressed only the environmental dimension, and studies that did consider both dimensions were only found for other constructive solutions not directly related to the case study analysed in this work, although it corresponds to a common practical application. This supports the comparison of the environmental and economic performances of alternative strengthening solutions for RC flexural members.

Concrete jacketing is the most traditional strengthening method when additional loads are introduced or seismic retrofitting is needed [13]. According to Thermou et al. [14], this method, despite being effective (if well executed), involves some issues due to the potential slippage between the existing structural member and the additional concrete layer. Moreover, the method involves the partial demolition of the member to be strengthened, requiring several labour-intensive tasks (namely, those necessary to guarantee monolithism) and is time-consuming, mostly due to the curing process of concrete. In addition, despite concrete still being one of the main materials used in construction [15], it also involves significant environmental impacts, due to the high demand for raw materials and the thermal processes required for cement production [16]. Efforts to reduce these impacts have been made, for instance, involving either the replacement of natural aggregates with recycled ones [16,17] or the development of alkali-based binders that might be able to substitute cement [18]; however, these developments still have low large-scale applicability. Hence, most concrete structures continue to have a significant environmental footprint.

Bonding a steel plate to a structural member is also a typical structural strengthening solution [19], being a less intrusive technique compared to concrete jacketing, since it only requires the (light) roughening of the surface of the member to be strengthened. However, this technique also presents some issues, mainly associated with the bonding agent, usually an epoxy adhesive [20,21], that may hinder the success of the solution depending on the quality control of the application process. Due to the significant weight of the steel plate, during the curing process of the adhesive, a shoring system is necessary to maintain pressure between the steel plate and the structural member [20]. Moreover, steel is prone to corrosion, thus an anti-oxidation paint is necessary to extend the service life of this solution.

The use of FRP strips in structural strengthening is analogous to bonding steel plates, and hence it also presents the same issues associated with adhesive bonding [22,23]. The main advantages of FRP composites are their high durability (non-corrodibility) and low maintenance [24], together with their high strength and lightness, which do not require a shoring system during the curing of the adhesive. Presently, due to these advantages, FRPs are often regarded as a more competitive solution than steel plates for many applications. The use of epoxy resins (and adhesives) in FRP composite solutions, has the consequence of presenting potentially significant cradle-to-gate environmental impacts, due to petroleum dependency; this problem is being addressed through the development of bio-based alternatives (e.g., [25]) that can decrease the FRPs’ environmental impacts in the future. Moreover, carbon fibres used in CFRP strips, require a significant amount of energy in production. In spite of their non-corrodibility and of empirical evidence of improved performance in harsh environments, FRP strengthening systems are still affected by various environmental effects, such as moisture and temperature [26,27,28]; in fact, the durability design of FRP systems is still an open research issue.

As mentioned, very limited information is available about the environmental and economic LCA of alternative rehabilitation or strengthening solutions. The most relevant studies found in the literature on these aspects are the ones by Palacios-Munoz et al. [29] and Demertzi et al. [30].

Palacios-Munoz et al. [29] performed environmental LCA of four strengthening solutions for RC beams: (i) anchored steel plate (without epoxy bonding); (ii) epoxy-adhered steel plate; (iii) epoxy-adhered CFRP strips; and (iv) concrete jacketing. This paper assessed the problem generically, with the objective of developing a model to predict the environmental impacts in equivalent kilograms of carbon dioxide (via the Global Warming Potential—GWP), and in megajoules (using the Abiotic Depletion of Resources—Fossil Fuels—ADP(ff) indicator). The model developed in that study allowed us to calculate the environmental impacts (GWP and ADP(ff)) depending on the geometry of the beam, the quality of concrete, the flexural strengthening ratio and the strengthening solution enabling the consideration of those parameters on the design process. Due to its generic nature, aiming to create an all-encompassing model, only two of the main environmental indicators were considered, and the economic dimension of the problem was disregarded. Hence the current study, focusing on one specific RC beam, but considering more environmental indicators, as well as the economic dimension, can provide further insights into the impacts of each strengthening solution.

Demertzi et al. [30] developed an environmental and economic LCA of five different floor rehabilitation systems for old buildings: (i) timber floor; (ii) beam-and-block floor; (iii) RC floor; (iv) steel–concrete composite floor; and (v) GFRP sandwich panels. The environmental and economic impacts were quantified for all solutions, including the transformation of the environmental impacts into economic prevention costs (externalities) through the eco-cost methodology [31], so that the material and labour costs could be summed to the environmental impacts’ externalities (eco-costs), hence allowing to rank the solutions. The study showed that, overall, the timber floors were the best-performing solution, with the GFRP sandwich panels being the worst (or second worst) in the environmental and economic (eco-cost) dimensions. In the present study, the eco-costs approach can provide relevant insights into the relative performance of each flexural strengthening solution.

## 3. Materials

### 3.1. Characteristics of the RC Beam

This study addresses four strengthening solutions, designed to increase the flexural strength of an existing RC beam by about 40%, a typical target value in building rehabilitation projects. The original RC beam has a cross-section of 30 cm (height) × 20 cm (width), a total length of 4.0 m, and a free span of 3.6 m. The beam is made of concrete class C25/30 and A500NR SD steel rebars (yield stress of 500 MPa and ultimate tensile stress of 575 MPa, according to LNEC E460:2017 [32]); the top and bottom longitudinal reinforcement consist, respectively, of two 8 mm and two 10 mm rebars; the transverse reinforcement consists of 8 mm stirrups with 10 cm of spacing.

### 3.2. Strengthening Methods

**Figure 1:**
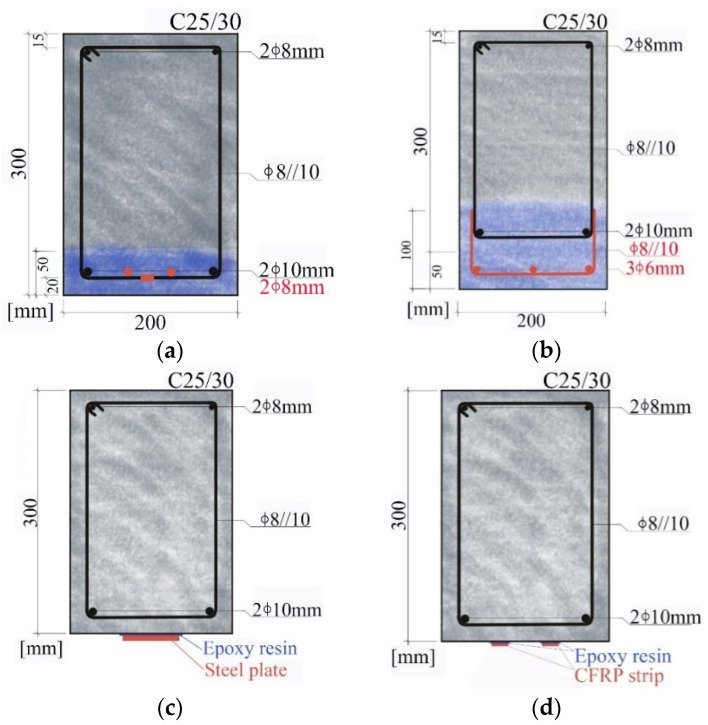
Illustration of the different strengthening methods: (**a**) CJE; (**b**) CJI; (**c**) ASP; (**d**) ACF.

The following four strengthening methods, illustrated in Figure 1, are considered and designed to provide a flexural strength increase of 40% (as close to as possible, as detailed ahead); the different solutions were designed following well-established calculation methodologies (e.g., those recommended in fib Bulletin 90 [33]), assuming that plane sections remain plane after bending and perfect bond between the concrete substrate and the strengthening materials:

- Concrete jacketing, by adding longitudinal rebars and keeping the cross-section unchanged (CJE);
- Concrete jacketing, by increasing the cross-section height, adding longitudinal rebars and extending the stirrups legs (CJI);
- Bonding a steel plate to the bottom soffit of the beam and keeping the cross-section unchanged (ASP);
- Bonding two CFRP strips to the bottom soffit of the beam and keeping the cross-section unchanged (ACF).

#### 3.2.1. Concrete Jacketing with Addition of Rebars (Method CJE)

The first strengthening solution involves adding two longitudinal 8 mm A500NR SD steel rebars to the existing cross-section, whose overall dimensions are kept unchanged (this can be an architectural requirement). This additional steel reinforcement provides a flexural strength increase of about 43% (corresponding to flexural failure due to concrete crushing after yielding the existing and the added steel rebars). Figure 1a illustrates this solution, in which the concrete that is demolished and reconstructed is represented in blue, while the new rebars and the welded stirrups are represented in red.

The following tasks are considered for the quantification of environmental and economic impacts: (i) removal of a 5 cm-thick layer of concrete from the bottom part of the section, exposing the existing steel reinforcement and creating space to apply the additional longitudinal rebars; (ii) cleaning of the (existing) steel rebars and exposed concrete; (iii) cutting of the stirrups with a manual steel guillotine or bolt cutters; (iv) applying the new longitudinal rebars; (v) welding the stirrups back together; (vi) applying steel wire to fix the new rebars in place; (vii) applying the formwork with reusable parts and release agent; (viii) pouring the concrete manually; (ix) removing the formwork.

#### 3.2.2. Concrete Jacketing with Height Increase and Addition of Rebars (Method CJI)

This solution is a variant of the previous one: besides the inclusion of three 6 mm A500NR additional bottom rebars (with 2 cm of cover), it involves increasing by 5 cm the height of the cross-section (to 20 × 35 cm^2^) and extending the legs of the stirrups (8 mm bars spaced by 10 cm). This allows for increasing the flexural strength by about 45% (corresponding to flexural failure due to concrete crushing after yielding of the existing and of the added steel rebars).

As in the previous solution, several tasks need to be carried out, namely: steps (i) and (ii) mentioned in Section 3.2.1; (iii) preparing (cutting with bolt cutters and bending manually) “U” shaped 8 mm rebars for extending the stirrups; (iv) mounting the additional longitudinal rebars with the stirrups extensions, using steel wire; (v) welding the additional (pre-fabricated) steel reinforcement to the existing one, directly on the beam (two welds per stirrup); (vi) applying the formwork (which is reusable) with release agent; (vii) pouring the concrete manually; (viii) removing the formwork.

This solution is illustrated in Figure 1b: the new concrete is represented in blue, including both the concrete that is demolished and the additional height, while the new steel reinforcement is represented in red, namely the additional longitudinal rebars and the stirrup extensions.

#### 3.2.3. Steel Plate Bonding (Method ASP)

This solution is less intrusive than the previous ones, requiring only the preparation of the concrete surface (roughening) where the plate is bonded. The solution designed in this study consists of applying a 70 mm wide, 3 mm thick and 3.5 m long S275JR steel plate, Figure 1c, and it allows for increasing the flexural strength by about 43% (corresponding to flexural failure due to concrete crushing after yielding of the existing steel rebars and of the bonded steel plate).

The application of this solution involves the following tasks: (i) roughening of the beam’s soffit with a light electric hammer that removes the superficial concrete layer (about 4 mm); (ii) cleaning the concrete surface with compressed air; (iii) manually applying epoxy adhesive to both the steel plate and the roughened concrete substrate (4 mm); (iv) manually applying the plate to the bottom soffit of the beam; (v) supporting the plate with a shoring system; (vi) removing the shoring system; and (vii) applying a protective (anti-corrosion) paint to the steel plate.

#### 3.2.4. CFRP Strip Bonding (Method ACF)

The strengthening solution based on bonding CFRP strips has an analogous procedure to the one used for steel plate bonding. In this case, the solution designed to strengthen the beam consists of bonding two CFRP strips to the beam’s soffit, both with 20 mm of width, 1.4 mm of thickness and 3.5 m of length, as adopted in Machado et al. [34] (Figure 1d). The CFRP strips have an elastic modulus of 170 GPa and tensile strength of 2890 MPa (values provided by the manufacturer [35]). This allows for increasing the flexural strength by about 41% (corresponding to failure due to CFRP debonding at a design strain of 0.46%).

The application procedure involves the steps (i) to (iii) mentioned in Section 3.2.3, followed by the manual application and bonding of the CFRP strips to the beam’s soffit. Due to the lightness of CFRP, a shoring system is not needed during the curing of the epoxy adhesive. The application of a protective paint was also disregarded due to the (well-known) non-corrodibility of CFRP systems.

## 4. Methods

### 4.1. Environmental LCA

#### 4.1.1. Scope

The environmental LCA methodology is an established and standardized methodology that aims to quantify, in an objective way, the environmental impacts embodied in a product or task [36,37]. The environmental impacts are calculated based on the generic (for all industries) international standards ISO 14040:2006 [38] and ISO 14044:2016 [39], with sectorial interpretations, such as in the construction sector: the European standard series EN 15643, in particular EN 15643-2:2011 [40] and EN 15804:2012+A2:2019 [41], which standardize the information presented in environmental product declarations (EPD) for construction products.

#### 4.1.2. Boundary

**Figure 2:**
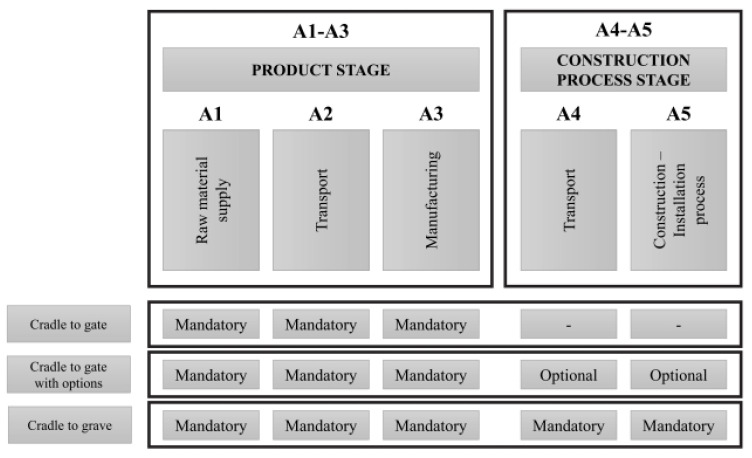
Product stage and construction process (adapted from EN 15804:2012+A2:2019 [31]).

As for the systems boundary considered in this study, and as defined in EN 15804:2012+A2:2019 [41], cradle-to-gate (C2G) plus optional A5 boundary (Figure 2) is considered in the LCA methodology to quantify the environmental impacts. Thus, the LCA stages considered are A1, A2, A3 and A5 which represent the following: A1—the extraction of raw materials; A2—transportation to factory; A3—transformation; and A5—application at construction site [41]. The product stage, specially the A1–A3 sub-stages, generally represent the majority of the impacts [42], being a good indicator of the whole life cycle of the considered solutions. The A4 stage, which represents transportation from the manufacturer to the construction site, is not considered, as it is not possible to define the methods of transportation, nor the distance travelled by the products in a theoretical case study, such as this one. Note that extending the analysis to the use stage (and end of life stage) would require detailed knowledge about the long-term durability of the various rehabilitation solutions, which is not yet available.

#### 4.1.3. Functional Unit

This study focuses on the application of alternative strengthening solutions to a specific case study, thus, the results are presented for the total environmental impacts of each specific solution, considering the whole length and total material and labour needed to achieve the ~40% threshold of flexural strength increase in the existing RC beam. Thus, the functional unit is defined as follows: a strengthening solution that allows increasing the flexural strength by, at least and as close to 40%, of an RC beam with 3.6 m of free span, initial cross-section of 20 × 30 cm^2^, two 8 mm and two 10 mm rebars as top and bottom reinforcement, respectively, and 8 mm stirrups spaced 10 cm apart as transverse reinforcement.

#### 4.1.4. Tasks and Quantities

For the development of this work, it is necessary to define the tasks that have to be carried out during the execution of the different strengthening solutions, and the amount of work and materials needed. To do so, a specialized prices generator website (Gerador de Preços) is consulted [43]. This website uses a national (Portuguese) database to identify the amount of work, different tasks, materials and labour needed to accomplish a given task. This way, it is possible to identify the tasks needed for each solution, and the quantity of each material and labour. The following specific materials are considered (instead of generic materials) and their characteristics are used: yield of anti-oxidant paint, epoxy resin and release agent; energy consumption of electric hammers, and soldering machine.

#### 4.1.5. Environmental Data

Regarding the environmental impacts, when possible, EPDs are used for the materials, with the objective of considering the most realistic and specific environmental impacts. All EPDs considered follow the standard EN 15804:2012 + A1:2013 [44]. These EPDs are used to the detriment of the more recent EN 15804:2012 + A2:2019 [45], due to the low number of EPDs following the latest version of this standard (which, at the time of this research, creates difficulties in finding products with the impact indicators accounted according to the same methods and units). When EPDs are not available, the software SimaPro version 9.4 [46] is used to search generic processes in the Ecoinvent V3.6 database [47], using the CML-IA baseline V3.06 method to calculate the missing environmental impacts.

In this study, the seven core environmental indicators are considered to estimate the environmental impacts of the different strengthening solutions, according to the standard EN 15804:2012 + A1:2013 (Table 1) [44].

### 4.2. Economic LCA

As for the economic assessment, the previously mentioned prices generator [43] is used to determine average market costs (in Portugal) for the considered tasks, as well as for the materials, at the date of this study. When possible, this information is complemented with real information from the manufacturers or suppliers of construction materials (e.g., steel [48] and electric energy prices).

### 4.3. Multicriteria Analysis

#### 4.3.1. Scope

The decision process of selecting among different construction products is complex, and different stakeholders might value different indicators and different characteristics of alternatives in very distinct ways. Since a reliable single score to rank environmentally the different solutions does not yet exist, and with the need to consider several environmental indicators, the development of a multicriteria analysis comprising environmental end economic costs adds value, by facilitating the data interpretation by non-specialists. In this work, the multicriteria analysis is achieved with two distinctive approaches: (i) a sensitivity analysis; and (ii) the eco-cost methodology, both briefly described in the next subsections.

#### 4.3.2. Sensitivity Analysis

With the calculation of the environmental and economic impacts of the four strengthening solutions, it is possible to normalize the achieved values into a scale of zero to one ([0, 1] interval). This normalization takes place by reducing the individual indicators to normalized figures, where “0” represents a null contribution (the normalized value of the indicator is zero) and “1” represents the maximum value of the indicator across all solutions. This aims to compare, in a relative manner, the alternatives considered. For example, GWP has a maximum value of 4.06 × 10^1^, thus this value is normalized to one and the other values are proportionally distributed between zero and one, considering that zero represents a GWP of 0.00 kg CO_2_ eq.: [0, Maximum Indicator Value] is transformed proportionally into [0, 1].

Having established an equal scale for all indicators, it is then possible to complete a sensitivity analysis (by attributing different weights to environmental and economic performance) to compare the four alternatives. However, there are still seven environmental indicators and a single economic one, thus potentially unbalancing the sensitivity analysis, since most decisions will depend on a balance between the economic and the environmental impact as a whole, and not on the value of single environmental impact indicator.

As there is not a standardized weighing method for the environmental indicators (many methods exist from different sources [49]), this study chose to consider that all environmental indicators have the same weight: hence, the total environmental impact of each solution results from the average of the normalized values (sum of the normalized values divided by the number of indicators).

This sensitivity analysis aims to provide an easy and pragmatic way to compare the strengthening solutions in their environmental and economic dimensions, by giving different weights to each of them; thus, helping stakeholders to decide, based on their environmental and economic requirements, which is the solution that best adjusts to their needs.

#### 4.3.3. Eco-Cost Methodology

The eco-cost methodology aims to quantify the costs of the environmental emissions, monetizing the prevention cost of the offset (cost to re-capture the emissions created) [50]. It is considered to provide “a measure to express the amount of environmental burden of a product on the basis of prevention of that burden” [31]. This facilitates the internalization of the externalities (indirect costs that are not reflected in the price paid), thus providing a tool to compare in an environmental and economic manner the different solutions by summing the direct economic costs and the eco-costs.

## 5. Results

### 5.1. Overview

This section presents the environmental and economic impacts of the four strengthening alternatives, including the sources of the data, quantities, and all relevant information that influences the results.

Firstly, the results are presented with the A1–A3 stages separated from the A5 stage; hence, in the first approach, the embodied environmental impacts in these stages are highlighted, followed by the impacts in the A5 stage, in which the environmental impacts result from a scenario that might vary with changes in the application procedures relative to those described previously.

The materials needed for each solution are presented and every scenario assumed is described. The sources for the data are also disclosed. To obtain the values presented, beyond the volumes, areas or weights considered, a 10% increase coefficient is applied to account for material waste during the construction process.

To calculate the environmental impacts of all solutions, the declared unit of each material is considered. This declared unit is, in most cases, the kilogram (kg), in the remaining cases it is volumetric (m^3^), so the materials density is used to transform it into kilograms, which is the unit used in the calculations. The functional unit considered for the presentation of the results is described in Section 4.1.3.

### 5.2. Product Stage (A1–A3)

The calculations for the product stage depend on the materials used and their quantities, followed by finding the sources of the environmental impacts (Table 2).

The following environmental sources are considered: (i) for concrete, an EPD for fresh concrete with the reference S-P-05207 [51]; (ii) for steel rebars, an EPD for steel reinforcing rebars with the reference S-P-05173 [52], (iii) for the steel wire, an EPD for hot rolled steel wire in a coil with the reference S-P-04744 [53]; and (iv) for the anti-corrosion coating, the EPD for Jomatastic 90 with the reference NEPD-2396-1134-EN [54]. With this information, it is then possible to calculate, for each of the solutions, the environmental impacts in the A1–A3 stage, considering the environmental factors referred to in Table 2; the results obtained are presented in Table 3.

### 5.3. Installation Stage (A5)

For the installation stage, two groups of assumptions are made for: (i) the concrete jacketing methods (CJE and CJI); and (ii) the plate bonding methods (ASP and ACF).

For the concrete jacketing methods, it is considered that the only tasks that have an impact are the demolition of the bottom part of the section, necessary to expose the steel reinforcement, and the welding of the stirrups after the application of the longitudinal rebars. The remaining activities are carried out with manual tools, not consuming any materials or energy that could have any sort of meaningful environmental impact. The following activities are not considered:

- Cutting the stirrups—executed with a manual bolt cutter;
- Bending the stirrups—by hand;
- Application of the longitudinal rebars—by hand;
- Application of formwork—by hand (materials with high number of reuses);
- Application of concrete—by hand;
- Removal and cleaning of formwork—by hand.

Thus, the tasks considered to have an environmental impact in the installation stage and the considerations assumed for the calculations are the following:

- Demolition: an electric hammer with a power of 1100 W, similar to a demolishing electric hammer 5.1 kg Makita HM870C (produced in Aichi, Japan), with an input of 0.072 m^3^/h, is considered.
- Welding: a machine similar to an Inverter Styl 1900 (produced in Girona, Spain) with a power of 6200 W, and 30 s per connection welded, is considered.

The construction demolition waste (CDW) generated and its disposal is not considered.

For the plate bonding methods, most of the tasks are manual, hence they do not involve environmental impacts. The only task that has environmental impacts is the surface roughening. The surface preparation is carried out with an electric hammer, similar to the demolition hammer considered before with only a different accessory in order to provide a light surface roughening:

- Surface preparation: the use of an electric hammer with a power of 1100 W, similar to a Makita demolishing electric hammer 5.1 kg HM870C, with an input of 1.49 m^2^/h, is considered.

The CDW generated by the surface roughening, with volumes of 7.35 × 10^−4^ m^3^ and 2.10 × 10^−4^ m^3^ for the ASP and ACF methods, respectively, is not considered relevant.

With the previous considerations, it is then possible to present the environmental impacts of the installation stage (A5) of the four methods (Table 4). The installation stage represents a much lower impact than the product stage. Considering the average across the seven environmental indicators, the installation stage represents 4.1% and 2.7% of the total environmental impacts for the concrete jacketing solutions, without and with height increase, respectively. For both plate bonding techniques, this figure is only 0.5%, which reflects the low amount of work required in the installation stage of those solutions.

### 5.4. Economic Impact

With all the calculated environmental impacts presented, it is then important to consider the economic cost of the four methods (Table 5). This analysis does not consider the total duration of the interventions, which may involve additional costs (e.g., associated with the logistics of the job site). It also does not consider the costs incurred during service life, namely those associated with maintenance and repair operations. The results obtained show that the two concrete jacketing solutions are significantly more expensive than the plate bonding ones: concrete jacketing with height increase and the addition of steel rebars and CFRP strip bonding are, respectively, the most and the least expensive methods.

### 5.5. Discussion

The environmental impacts of each solution reflect those of the corresponding material components. Here, the relative impacts of the components are presented with a brief explanation of why some materials contribute more to a given environmental impact.

**Figure 3:**
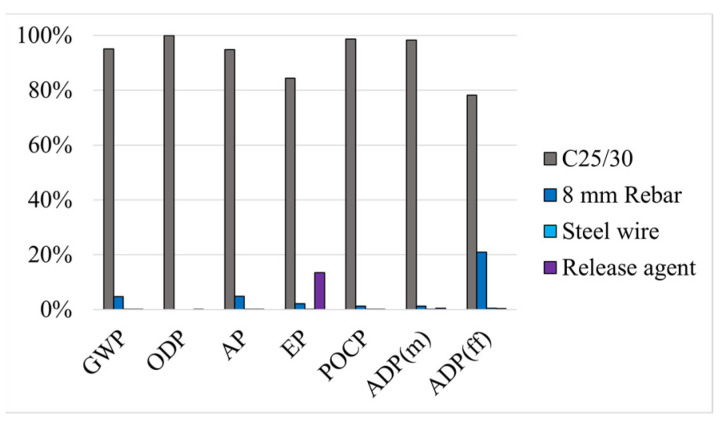
Method CJE: relative environmental impacts of each material.

**Figure 4:**
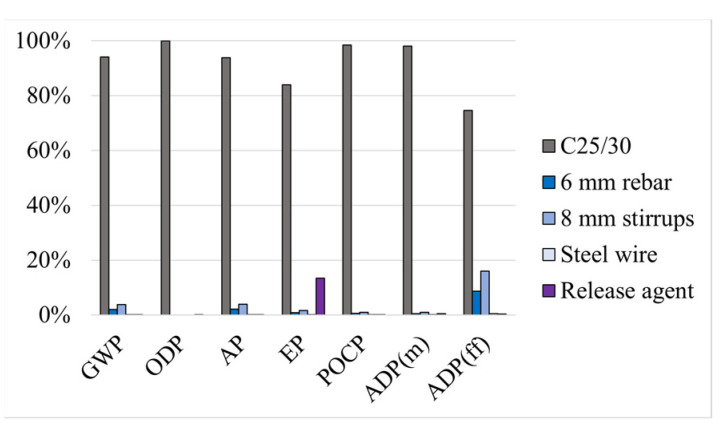
Method CJI: relative environmental impacts of each material.

First, and analysing the two solutions that use concrete to strengthen the RC beam, solution CJE (Figure 3) and solution CJI (Figure 4), it is possible to observe that the addition of the concrete (C25/30) layer has the highest contribution to the overall environmental impacts, whatever the impact category considered. This reflects the high environmental impact of concrete, due to the use of cement. In fact, cement alone contributes to about 7% of all GHG emissions produced by humans worldwide [4], due to the calcination of limestone, which is a high energy consumption process [55]. Also, in both solutions, the rebars (with diameters of 6 or 8 mm) present relatively high contributions in GWP and ADP(ff), which can be explained by the use of energy during the manufacturing of steel. In addition, the release agent, despite being used only in small quantities, presents a high contribution to the total impact in the EP category; this is explained by the source material of the release agent, paraffin, which involves high consumption of energy from non-renewable sources (according to the considered process).

**Figure 5:**
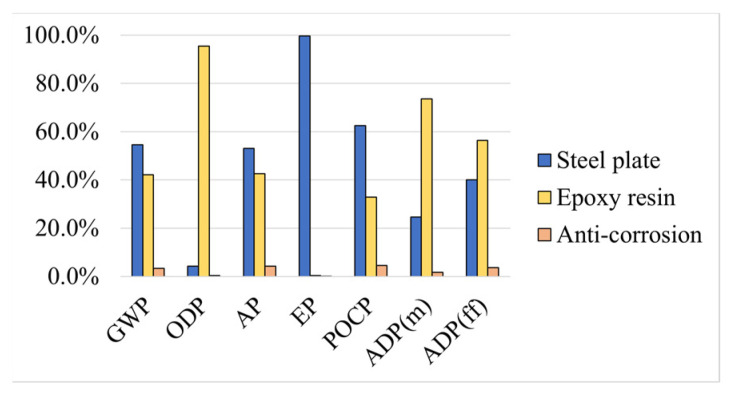
Method ASP: relative environmental impacts of each material.

**Figure 6:**
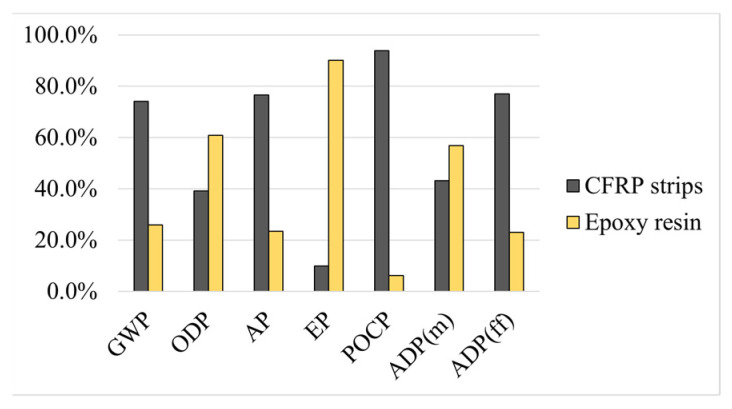
Method ACF: relative environmental impacts of each material.

Regarding the plate bonding solutions, solution ASP (Figure 5) and solution ACF (Figure 6) involve the use of epoxy adhesive, which presents high impacts in almost all categories. However, in EP, the epoxy adhesive presents a negligible contribution to the total impact of ASP, but a high relative impact to ACF, which is due to the fact that steel has a high impact in EP (due to the consumption of energy from non-renewable sources) and carbon fibres have a very low impact there. Therefore, the ACF solution has a very low impact but almost 90% of that impact in the EP category comes from the epoxy adhesive.

## 6. Multicriteria Analysis

### 6.1. Impact Normalization

#### 6.1.1. Data Summary

The discussion presented next, in line with the scope of this study, is first focused on the comparison between the environmental and economic impacts of the different solutions, considering the full boundary assumed (A1–A3+A5). The total impacts, both environmental and economic, are presented in Table 6, with their normalization (as explained in Section 4.3.2) presented next in Table 7.

In this sensitivity analysis, it is assumed that, for most stakeholders, there are two main variables: (i) cost; and (ii) environmental impact. Hence, to simplify the analysis, the seven environmental indicators were aggregated into a single score. Considering the same importance for all the environmental indicators, this single score was defined as the sum of all the normalized indicators. Accordingly, Table 7 also presents the sum of all the normalized environmental indicators per solution (Ʃ column). Table 8 lists three indicators: (i) Envt, calculated by dividing Ʃ by the number of indicators (seven); (ii) EnvtN, which is the Envt indicator normalized in the interval [0, 1] relative to the highest value of Envt obtained here; (iii) Cost, which, as for the environmental indicators, is calculated through a normalization comprised in the interval [0, 1] relative to the highest cost determined among the four strengthening solutions.

This analysis shows that the strengthening method that involves bonding a steel plate to the soffit of the RC beam (ASP) has the greatest environmental impact overall, followed closely by the method of bonding CFRP strips (ACF). The lowest environmental impact is obtained by concrete jacketing without increasing the beams’ section (CJE), followed by the concrete jacketing with section increase (CJI). In terms of costs, the concrete jacketing method with increased section has the highest impact, followed very closely by the same method without an increase in section. The other two methods present economic impacts of about half the CJE solution, with the bonding of a steel plate (ASP) or CFRP strips (ACF) presenting economic impacts of, respectively, 59% and 46% of the CJI solution.

#### 6.1.2. Sensitivity Analysis

Since different stakeholders value information differently, especially environmental, and economic impacts, a sensitivity analysis that allows to compare the performance of the solutions varying the weight of the normalized environmental (EnvtN) and economic (Cost) impacts can be a useful tool to support decisions. To perform such sensitivity analysis, the weight of the two indicators studied varies in steps of 0.1 (or 10%). The results of this analysis are presented in Table 9.

This sensitivity analysis divides the choice of an optimum solution into three groups:

- EnvtN ϵ [1.0, 0.6] Ʌ Cost ϵ [0.0, 0.4]: in this range, the EnvtN indicator is dominant, meaning that the solutions rank exactly as their EnvtN indicator; CJE is the best solution, followed by CJI and ACF, with the worst solution being ASP;
- EnvtN ϵ [0.5, 0.3] Ʌ Cost ϵ [0.5, 0.7]: in this interval, the relative rank of the methods is not always the same, with the best solutions varying from CJE to ACF when the environmental indicator becomes less important;
- EnvtN ϵ [0.2, 0.0] Ʌ Cost ϵ [0.8, 1.0]: the Cost indicator becomes dominant, and the solutions rank exactly as the Cost indicator; ACF is the best solution, followed by ASP and CJE, and the worst solution is CJI.

In summary, when the stakeholders solely or mostly focus on environmental impacts, then the concrete jacketing solution CJE presents the best performance; however, when the cost is more valued, then the CFRP strip bonding solution ACF presents the best overall performance. It is worth remembering that this analysis excluded the service life and end-of-life stages of the strengthened RC members, for which different environmental and economic impacts are expected among the four solutions due to their different durability and maintenance requirements—future studies should address these aspects.

### 6.2. Eco-Cost Methodology

The eco-costs represent a cost to avoid or counter emissions related to five environmental impact categories: GWP; ODP; AP; EP; and POCP. In Table 10, the eco-costs per kilogram of equivalent emission are presented for each environmental indicator, followed by the eco-costs per strengthening solution [31].

The two remaining indicators, ADP(m) and ADP(ff), are not listed for different reasons: ADP(m) represents the value of avoiding the depletion of mineral resources, which varies depending on the mineral being depleted, thus being difficult to define; ADP(ff) is already indirectly considered in GWP, as most of the carbon dioxide emissions are originated through the burning of fossil fuels; thus, according to standard ISO 14044:2006 [39], which determines that the double counting of impacts shall be avoided, ADP(ff) does not have a monetization of externalities [31].

With this information, it is then possible to assess the total cost of each solution, considering the direct cost of materials and labor, as well as the environmental externalities (Table 11).

The eco-costs represent a small contribution to the total cost of each solution (1.1% to 2.4%), with the exception of steel plate bonding (ASP), for which the eco-cost contributes nearly 12% of the total final cost due to the high EP emissions from the manufacturing of steel plates due to the consumption of non-renewable energy.

With the consideration of costs and eco-costs, the CFRP strip bonding solution (ACF) presents the overall lowest cost, followed by steel plate bonding (ASP). Both concrete jacketing solutions present higher costs. As mentioned, these results do not take into account the service life stage, during which maintenance and repair works are necessary, involving additional costs and environmental impacts. As mentioned, future research is required to enable an accurate consideration of the service life and end-of-life stages.

### 6.3. Eco-Cost vs. Sensitivity Analysis

Eco-cost and sensitivity analysis are methodologies that aim to solve the same issue: how to incorporate environmental indicators into a multi-criteria decision-making process. Thus, it is relevant to compare the results of both methodologies—this comparison is presented in Table 12 and Table 13. In Table 12, the eco-costs and the total costs are both normalized relative to the highest respective values determined in this study.

The comparative analysis summarized in Table 12 and Table 13 shows that the eco-cost methodology renders an almost equal result as the sensitivity analysis when only cost is considered. In fact, the consideration of the eco-cost makes almost no difference for three of the four methods studied. It is only for the steel plate bonding method (ASP) that the eco-cost solution is not similar to the sensitivity analysis that only considers the economic cost; for this solution, results from both approaches match for an environmental weight in the interval [0.1, 0.2].

## 7. Conclusions

This study was developed with the objective of comparing different flexural strengthening solutions for an RC beam using environmental and economic cradle-to-gate LCA methodologies.

The multicriteria and sensitivity analysis developed in this study shows that concrete jacketing keeping the cross-section of the RC beam unchanged presents the best overall performance if stakeholders value the environmental impacts more than economic impacts (normalized environmental impacts weighed at factor 0.4 or higher). The lowest cost of the CFRP strip bonding solution is a key advantage when cost is a determinant factor for the decision process, which is very often the case.

The eco-cost approach presents similar results to the sensitivity analysis when only the cost is considered for all strengthening methods, except for steel plate bonding; for this latter method, similar results are obtained when, in the sensitivity analysis, environmental impacts are considered with a weight of 0.1 to 0.2. Hence, especially the steel plate bonding solution is highly affected by the eco-cost approach, as its eutrophication emission value is very high because the cost associated with avoiding and/or off-setting this emission is high, this strengthening solution presents the highest eco-cost among the alternatives evaluated. This is particularly obvious when comparing both bonding methods: the externalities of the CFRP strip and steel plate bonding solutions are, respectively, EUR 2.99 and EUR 21.62.

In the sensitivity analysis, when the environmental factor is dominant (normalized environmental impacts weighed equal to or higher than 0.6), both concrete jacketing solutions are the best-performing ones—the one that keeps the section constant is the best because it consumes less materials. When the relevance of the cost factor increases (weight equal to or higher than 0.8), the bonding solutions become the most attractive, with the CFRP strips bonding being the best-performing one. When the cost and environmental factors present similar weights (normalized environmental impacts weight between 0.5 and 0.3 and cost weight between 0.5 and 0.7), the four methods, despite being very different, present fairly similar scores, with maximum differences between the best and the worse performing solutions varying between 0.21 and 0.25. These figures compare with maximum differences of 0.96 and 0.54 when the normalized environmental impacts weight is 1.0, i.e., when the decision is based only on environmental impacts.

This study was developed considering a specific (but typical) strengthening case study, comparing four different solutions often used in engineering practice. The extension of the findings to other strengthening interventions (e.g., confinement of reinforced concrete columns) should be investigated in future studies. Moreover, it should be highlighted that only the product stage (A1–A3) was considered, as most environmental impacts are concentrated in this stage [56]. Considering the whole life cycle will add value to the decision-making, especially if maintenance operations prove to be relevant for one or more solutions. Thus, future works on the durability of the strengthening solutions (namely the ones involving bonding operations and the use of FRP systems) and their impact on maintenance and life cycle assessment will add valuable insights and inform future strengthening decisions.

## Figures and Tables

**Table 1:** Environmental indicators considered.

Parameter	Abbreviation	Unit
Global Warming Potential	GWP	kg CO_2_ eq.
Ozone Depletion Potential	ODP	kg CFC11 eq.
Acidification Potential	AP	kg SO_2_ eq.
Eutrophication Potential (freshwater)	EP	kg PO_4_ eq.
Photochemical Ozone Formation	POCP	kg C_2_H_4_ eq.
Abiotic Depletion of Resources (mineral)	ADP(m)	kg Sb eq.
Abiotic Depletion of Resources (fossil fuels)	ADP(ff)	MJ

**Table 2:** Materials, quantities, and sources for environmental data.

Method	Material		Quantity		Total	Source	
	Type	Sub-Type			[kg]	Type	Code/Process
CJE	Concrete	C25/30	0.040	m^3^	99.00	EPD	S-P-05207
	Rebar	8 mm A500NR	2.772	kg	2.772	EPD	S-P-05173
	Steel wire	A500NR	0.053	kg	0.053	EPD	S-P-04744
	Release agent	Petroleum base	0.005	l	0.004	Ecoinvent V3.6	(1)
CJI	Concrete	C25/30	0.079	m^3^	198.0	EPD	S-P-05207
	Rebar	6 mm A500NR	2.398	kg	2.398	EPD	S-P-05173
	Stirrups	8 mm A500 NR	4.435	kg	4.435	EPD	S-P-05173
	Steel wire	A500NR	0.107	kg	0.107	EPD	S-P-04744
	Release agent	Petroleum base	0.010	l	0.008	Ecoinvent V3.6	(1)
ASP	Steel plate	S275 JR	5.770	kg	5.770	Ecoinvent V3.6	(2)
	Adhesive	Epoxy	1.568	kg	1.568	Ecoinvent V3.6	(3)
	Paint	Anti-corrosion	0.146	kg	0.146	EPD	NEPD-2396-1134-EN
ACF	CFRP Strip	Pultruded	0.314	kg	0.314	EcoCalculator	(4)
	Adhesive	Epoxy	0.896	kg	0.896	Ecoinvent V3.6	(3)

(1) Paraffin {GLO}| market for | Cut-off, S. (2) Steel, unalloyed {RER}| steel production, converter, unalloyed | Cut-off, S. (3) Epoxy resin, liquid {RER}| market for epoxy resin, liquid | Cut-off, S. (4) Assuming 0.28 kg of epoxy resin + 0.77 kg of carbon fibre per kg of CFRP (including material waste).

**Table 3:** Environmental impacts for the product stage (A1–A3).

Solution	GWP	ODP	AP	EP	POCP	ADP(m)	ADP(ff)
	kg CO_2_ eq.	kg CFC11 eq	kg SO_2_ eq.	kg PO_4_ eq.	kg C_2_H_4_ eq.	kg Sb eq.	MJ
CJE	2.01 × 10^+01^	3.90 × 10^−07^	2.53 × 10^−02^	1.62 × 10^−02^	6.68 × 10^−03^	1.55 × 10^−05^	6.96 × 10^+01^
CJI	4.06 × 10^+01^	7.80 × 10^−07^	5.13 × 10^−02^	3.25 × 10^−02^	1.34 × 10^−02^	3.11 × 10^−05^	1.46 × 10^+02^
ASP	1.83 × 10^+01^	1.27 × 10^−05^	7.03 × 10^−02^	3.99 × 10^+00^	1.18 × 10^−02^	1.87 × 10^−04^	2.46 × 10^+02^
ACF	1.70 × 10^+01^	1.14 × 10^−05^	7.32 × 10^−02^	7.72 × 10^−03^	3.61 × 10^−02^	1.38 × 10^−04^	3.45 × 10^+02^

**Table 4:** Environmental impacts for the installation stage (A5).

Solution	Energy		Source	GWP	ODP	AP	EP	POCP	ADP(m)	ADP(ff)
	[W]	[MJ]		kg CO_2_ eq.	kg CFC11 eq	kg SO_2_ eq.	kg PO_4_ eq.	kg C_2_H_4_ eq.	kg Sb eq.	MJ
CJE	3238.1	11.66	(5)	1.28 × 10^+00^	6.20 × 10^−08^	9.33 × 10^−03^	2.20 × 10^−03^	3.47 × 10^−04^	8.33 × 10^−06^	1.49 × 10^+01^
CJI	5098.1	18.35	(5)	5.46 × 10^−01^	2.64 × 10^−08^	3.97 × 10^−03^	9.38 × 10^−04^	1.48 × 10^−04^	3.55 × 10^−06^	6.35 × 10^+00^
ASP	356.4	1.28	(5)	1.41 × 10^−01^	6.83 × 10^−09^	1.03 × 10^−03^	2.42 × 10^−04^	3.82 × 10^−05^	9.17 × 10^−07^	1.64 × 10^+00^
ACF	194.8	0.70	(5)	7.71 × 10^−02^	3.73 × 10^−09^	5.61 × 10^−04^	1.33 × 10^−04^	2.09 × 10^−05^	5.01 × 10^−07^	8.98 × 10^−01^

(5) Electricity, low voltage {PT} | market for | Cut-off, S.

**Table 5:** Economic impacts for the considered methods.

Solution	Task	Partial	%	Total
CJE	Preparation	EUR 55.28	20.7%	EUR 266.41
	Reinforcement	EUR 211.14	79.3%	
CJI	Preparation	EUR 55.28	20.3%	EUR 272.88
	Reinforcement	EUR 217.60	79.7%	
ASP	Preparation	EUR 7.23	4.5%	EUR 160.27
	Reinforcement	EUR 153.04	95.5%	
ACF	Preparation	EUR 4.13	3.3%	EUR 124.22
	Reinforcement	EUR 120.09	96.7%	

**Table 6:** Summary of the total environmental and economic impacts of the various solutions.

Solution	GWP	ODP	AP	EP	POCP	ADP(m)	ADP(ff)	Cost
	kg CO_2_ eq.	kg CFC11 eq	kg SO_2_ eq.	kg PO_4_ eq.	kg C_2_H_4_ eq.	kg Sb eq.	MJ	EUR
CJE	2.14 × 10^+01^	4.52 × 10^−07^	3.47 × 10^−02^	1.84 × 10^−02^	7.02 × 10^−03^	2.39 × 10^−05^	8.45 × 10^+01^	EUR 266.41
CJI	4.26 × 10^+01^	8.77 × 10^−07^	6.59 × 10^−02^	3.60 × 10^−02^	1.39 × 10^−02^	4.42 × 10^−05^	1.69 × 10^+02^	272.88
ASP	1.84 × 10^+01^	1.28 × 10^−05^	7.13 × 10^−02^	3.99 × 10^+00^	1.18 × 10^−02^	1.88 × 10^−04^	2.48 × 10^+02^	160.27
ACF	1.71 × 10^+01^	1.14 × 10^−05^	7.38 × 10^−02^	7.86 × 10^−03^	3.61 × 10^−02^	1.39 × 10^−04^	3.46 × 10^+02^	124.22

**Table 7:** Normalized environmental indicators.

Solution	GWP	ODP	AP	EP	POCP	ADP(m)	ADP(ff)	Ʃ
CJE	0.50	0.04	0.47	0.00	0.19	0.13	0.24	1.58
CJI	1.00	0.07	0.89	0.01	0.39	0.24	0.49	3.08
ASP	0.43	1.00	0.97	1.00	0.33	1.00	0.72	5.44
ACF	0.40	0.90	1.00	0.00	1.00	0.74	1.00	5.04

**Table 8:** Normalized values.

Solution	Envt	EnvtN	Cost
CJE	0.02	0.04	0.98
CJI	0.36	0.53	1.00
ASP	0.68	1.00	0.59
ACF	0.66	0.96	0.46

**Table 9:** Sensitivity analysis.

EnvtN weight	1.0	0.9	0.8	0.7	0.6	0.5	0.4	0.3	0.2	0.1	0.0
Cost weight	0.0	0.1	0.2	0.3	0.4	0.5	0.6	0.7	0.8	0.9	1.0
CJE	0.04	0.13	0.22	0.32	0.41	0.51	0.60	0.69	0.79	0.88	0.98
CJI	0.53	0.58	0.62	0.67	0.72	0.76	0.81	0.86	0.91	0.95	1.00
ASP	1.00	0.96	0.92	0.88	0.83	0.79	0.75	0.71	0.67	0.63	0.59
ACF	0.96	0.91	0.86	0.81	0.76	0.71	0.66	0.61	0.56	0.51	0.46

**Table 10:** Quantification of eco-costs.

Indicator	GWP (EUR)	ODP (EUR)	AP (EUR)	EP (EUR)	POCP (EUR)	Total (EUR)
Per kg	0.116	120.00	8.75	4.70	9.08	Eco-cost
CJE	2.48	0.00	0.30	0.09	0.06	2.93
CJI	4.95	0.00	0.58	0.17	0.13	5.82
ASP	2.14	0.00	0.62	18.75	0.11	21.62
ACF	1.98	0.00	0.65	0.04	0.33	2.99

**Table 11:** Total cost and contribution considering the eco-cost methodology.

Solution	Cost		Eco-Cost		Total (EUR)
	[EUR]	%	[EUR]	%	
CJE	266.41	98.9%	2.93	1.1%	269.35
CJI	272.88	97.9%	5.82	2.1%	278.70
ASP	160.27	88.1%	21.62	11.9%	181.89
ACF	124.22	97.6%	2.99	2.4%	127.22

**Table 12:** Normalization of cost, eco-cost and total cost.

Solution	Cost	Eco-Cost	Total
CJE	0.98	0.14	0.97
CJI	1.00	0.27	1.00
ASP	0.59	1.00	0.65
ACF	0.46	0.14	0.46

**Table 13:** Reduced sensitivity analysis.

EnvtN weight	0.2	0.1	0.0
Cost weight	0.8	0.9	1.0
CJE	0.79	0.88	0.98
CJI	0.91	0.95	1.00
ASP	0.67	0.63	0.59
ACF	0.56	0.51	0.46

## Data Availability

The original contributions presented in the study are included in the article, further inquiries can be directed to the corresponding author.
